# Subjective Aging in Context: Neighborhood Social Environment and Self-perceptions of Aging among Older Adults

**DOI:** 10.1093/geroni/igab046.2316

**Published:** 2021-12-17

**Authors:** Eunyoung Choi, Elizabeth Zelinski, Jennifer Ailshire

**Affiliations:** University of Southern California, Los Angeles, California, United States

## Abstract

Self-perception of aging (SPA), one’s attitude toward one’s own aging, has been associated with health and well-being in later life. Whereas existing literature identifies individual-level predictors of SPA (e.g., education and health), little is known about the role of neighborhood context. The present study examines whether 1) neighborhood social environment is related to SPA and 2) age moderates this relationship. Our analytic sample includes 11,394 adults aged 50+ from the 2014 and 2016 waves of the Health and Retirement Study (Mean Age=68, SD=10.14, range 50-98). Indicators of neighborhood social environment include (a) perceived neighborhood social cohesion (the trust and social ties among community residents), (b) neighborhood friends, and (c) relatives living in the neighborhood. Regression analyses were performed to investigate the associations of each neighborhood-level indicator with the positive and negative dimensions of SPA. The models controlled for demographic, socio-economic, and health covariates. Greater neighborhood social cohesion (B=0.13, SE=0.01, p<.001) and having neighborhood friends (B=0.14, SE=0.02, p<0.001) were associated with higher levels of the positive SPA. As for the negative dimension of SPA, neighborhood social cohesion was the only significant predictor (B=-0.13, SE=0.01, p<0.001). Furthermore, we found significant interaction effects between neighborhood social cohesion and age: higher neighborhood cohesion was associated with more positive (B=-.003, SE=.00, p<.001) and less negative SPA ratings (B=-.003, SE=.00, p<.001) at younger ages than older ages. Our findings provide insights into how neighborhood social context shapes subjective aging, suggesting that a socially cohesive neighborhood may promote more favorable perceptions of aging, particularly for younger residents.

